# Periprosthetic Joint Infection After Total Knee Arthroplasty With or Without Antibiotic Bone Cement

**DOI:** 10.1001/jamanetworkopen.2024.12898

**Published:** 2024-05-23

**Authors:** Tesfaye H. Leta, Stein Atle Lie, Anne Marie Fenstad, Stein Håkon L. Lygre, Martin Lindberg-Larsen, Alma B. Pedersen, Annette W-Dahl, Ola Rolfson, Erik Bülow, Liza N. van Steenbergen, Rob G. H. H. Nelissen, Dylan Harries, Richard de Steiger, Olav Lutro, Keijo Mäkelä, Mikko S. Venäläinen, Jinny Willis, Michael Wyatt, Chris Frampton, Alexander Grimberg, Arnd Steinbrück, Yinan Wu, Cristiana Armaroli, Maria Adalgisa Gentilini, Roberto Picus, Mirko Bonetti, Serban Dragosloveanu, Andreea E. Vorovenci, Dan Dragomirescu, Håvard Dale, Christian Brand, Bernhard Christen, Joanne Shapiro, J. Mark Wilkinson, Richard Armstrong, Kate Wooster, Geir Hallan, Jan-Erik Gjertsen, Richard N. Chang, Heather A. Prentice, Art Sedrakyan, Elizabeth W. Paxton, Ove Furnes

**Affiliations:** 1 The Norwegian Arthroplasty Register, Department of Orthopedic Surgery, Haukeland University Hospital, Bergen, Norway; 2 Faculty of Health Science, VID Specialized University, Oslo, Norway; 3 Department of Population Health Sciences, Weill Medical College of Cornell University, New York, New York; 4 Medical Device Surveillance and Assessment, Kaiser Permanente, San Diego, California; 5 Center for Translational Oral Research, Department of Dentistry, University of Bergen, Bergen, Norway; 6 Department of Occupational Medicine, Haukeland University Hospital, Bergen, Norway; 7 The Danish Knee Arthroplasty Register, Odense, Denmark; 8 Department of Orthopaedic Surgery and Traumatology, Odense University Hospital, Odense, Denmark; 9 Department of Clinical Epidemiology, Aarhus University Hospital and Department of Clinical Medicine, Aarhus University, Aarhus, Denmark; 10 The Swedish Arthroplasty Register, Gothenburg, Sweden; 11 Department of Clinical Sciences Lund, Orthopedics, Lund University, Lund, Sweden; 12 Institute of Clinical Sciences, Sahlgrenska Academy, University of Gothenburg, Gothenburg, Sweden; 13 Centre of Registers Västra Götaland, Gothenburg, Sweden; 14 The Dutch Arthroplasty Register, ‘s-Hertogenbosch, the Netherlands; 15 Department Orthopaedics, Leiden University Medical Center, Leiden, the Netherlands; 16 South Australian Health and Medical Research Institute, Adelaide, Australia; 17 The Australian Orthopaedic Association National Joint Replacement Registry, Adelaide, Australia; 18 Department of Medicine, Stavanger University Hospital, Stavanger, Norway; 19 The Finnish Arthroplasty Register, Helsinki, Finland; 20 Turku University Hospital and University of Turku, Turku, Finland; 21 Department of Medical Physics, Turku University Hospital, Turku, Finland; 22 The New Zealand Joint Registry, Christchurch, New Zealand; 23 German Arthroplasty Registry, Berlin, Germany; 24 Arthroplasty Registry of the Autonomous Province of Trento, Clinical Epidemiology Service, Provincial Agency for Health Services of Trento, Trento, Italy; 25 Arthroplasty Register of Autonomous Province of Bolzano, Observatory of Health, Health Department AP of Bolzano, Bolzano, Italy; 26 Romanian Arthroplasty Registry, Bucharest, Romania; 27 University of Medicine and Pharmacy–Carol Davila, Bucharest, Romania; 28 Foisor Orthopaedic Hospital, Bucharest, Romania; 29 Economic Cybernetics and Statistics Doctoral School, Bucharest University of Economic Studies, Bucharest, Romania; 30 Department of Clinical Medicine, Faculty of Medicine, University of Bergen, Bergen, Norway; 31 Swiss National Hip and Knee Joint Registry, Bern, Switzerland; 32 Institute of Social and Preventive Medicine, SwissRDL, University of Bern, Bern, Switzerland; 33 Articon, Bern, Switzerland; 34 The National Joint Registry for England, Wales, Northern Ireland, The Isle of Man and Guernsey, London, United Kingdom; 35 NEC Software Solutions, Hemel Hempstead, United Kingdom; 36 Division of Clinical Medicine, School of Medicine and Population Health, University of Sheffield, Sheffield, United Kingdom

## Abstract

**Importance:**

Despite increased use of antibiotic-loaded bone cement (ALBC) in joint arthroplasty over recent decades, current evidence for prophylactic use of ALBC to reduce risk of periprosthetic joint infection (PJI) is insufficient.

**Objective:**

To compare the rate of revision attributed to PJI following primary total knee arthroplasty (TKA) using ALBC vs plain bone cement.

**Design, Setting, and Participants:**

This international cohort study used data from 14 national or regional joint arthroplasty registries in Australia, Denmark, Finland, Germany, Italy, New Zealand, Norway, Romania, Sweden, Switzerland, the Netherlands, the UK, and the US. The study included primary TKAs for osteoarthritis registered from January 1, 2010, to December 31, 2020, and followed-up until December 31, 2021. Data analysis was performed from April to September 2023.

**Exposure:**

Primary TKA with ALBC vs plain bone cement.

**Main Outcomes and Measures:**

The primary outcome was risk of 1-year revision for PJI. Using a distributed data network analysis method, data were harmonized, and a cumulative revision rate was calculated (1 − Kaplan-Meier), and Cox regression analyses were performed within the 10 registries using both cement types. A meta-analysis was then performed to combine all aggregated data and evaluate the risk of 1-year revision for PJI and all causes.

**Results:**

Among 2 168 924 TKAs included, 93% were performed with ALBC. Most TKAs were performed in female patients (59.5%) and patients aged 65 to 74 years (39.9%), fully cemented (92.2%), and in the 2015 to 2020 period (62.5%). All participating registries reported a cumulative 1-year revision rate for PJI of less than 1% following primary TKA with ALBC (range, 0.21%-0.80%) and with plain bone cement (range, 0.23%-0.70%). The meta-analyses based on adjusted Cox regression for 1 917 190 TKAs showed no statistically significant difference at 1 year in risk of revision for PJI (hazard rate ratio, 1.16; 95% CI, 0.89-1.52) or for all causes (hazard rate ratio, 1.12; 95% CI, 0.89-1.40) among TKAs performed with ALBC vs plain bone cement.

**Conclusions and Relevance:**

In this study, the risk of revision for PJI was similar between ALBC and plain bone cement following primary TKA. Any additional costs of ALBC and its relative value in reducing revision risk should be considered in the context of the overall health care delivery system.

## Key Points

**Question:**

What is the estimated risk of revision for periprosthetic joint infection (PJI) after total knee arthroplasty (TKA) using antibiotic-loaded bone cement (ALBC) vs plain bone cement?

**Findings:**

This cohort study of 2 168 924 cemented primary TKAs for osteoarthritis between 2010 and 2020 found no difference in risk of revision for PJI in TKAs with plain bone cement compared with TKAs with ALBC at 1 year.

**Meaning:**

These findings suggest that the routine use of ALBC in primary TKA should be considered in the context of the overall health care delivery system.

## Teaser

This cohort study uses data from 14 national or regional total knee arthroplasty (TKA) registries to compare the rates of revision owing to periprosthetic joint infection among patients who received antibiotic-loaded bone cement or plain bone cement.

## Introduction

Globally, the number of joint arthroplasties performed is increasing over time,^1,2,3,4,5,6^ with a parallel increase in the prevalence of periprosthetic joint infection (PJI). PJI following joint arthroplasty remains a devastating complication despite significant advances in perioperative antimicrobial procedures.^7,8^ Revision for PJI is the most frequent cause of revision after total knee arthroplasty (TKA).^9,10,11,12^ The cost burden of revision for PJI is more than twice that of non-PJI revision, and patients often require repeated surgery with poor outcomes.^13^ In the US, the mean total cost for revision TKA was $75 028.07, ranging from $42 915.63 for patellar component revision to $90 065.11 for femoral component revision.^14^ However, this cost differential may vary by country.

Over the last 50 years, antibiotic-loaded bone cement (ALBC) has been used as a prophylactic measure in joint arthroplasty to reduce the risk of PJI.^7,15^ In the Nordic countries, ALBC has been used as a standard prophylactic measure in primary arthroplasty for more than 2 decades.^7,16,17^ In some European countries and in North America, use of ALBC is controversial.^7,17,18,19^ From 2006 to 2016, the utilization rate of ALBC in primary TKA in the US was 27%.^18^ This low percentage of ALBC use may be related to the fact that ALBC is not approved by the US Food and Drug Administration in primary joint replacement (except for patients at high risk^17^) and related to the cost of ALBC compared with plain bone cement. A 2023 international multiregister study reported that ALBC use in primary TKA varies internationally, ranging from 31% in the US to 100% in Norway.^20^

Several investigations have suggested the addition of antibiotics to bone cement has disadvantages, such as antimicrobial resistance,^17,21,22,23^ with a subsequent increase in health care costs.^17,24,25^ Support for the use of ALBC in the literature, including efficacy in reducing revision for PJI, is limited.^26,27,28,29^ The International Consensus Meeting on PJI in 2018 demonstrated no consensus on the routine use of ALBC in primary total hip or knee arthroplasty with the aim of reducing the risk of subsequent PJIs.^30^ Earlier studies on use of ALBC in primary arthroplasty have called for large, prospective, and preferably multicenter studies to justify routine use.^20,31,32^

Using a multiregistry meta-analysis approach, the primary aim of this study was to assess the association of ALBC use in primary TKA in the risk of 1-year revision for PJI. The secondary aim was to assess the risk of revision for PJI and all causes following primary TKA with ALBC compared with plain bone cement at 3-month and 1-, 5-, and 10-year follow-ups.

## Methods

This study was initiated by the Norwegian Arthroplasty Register (NAR), and NAR coordinated the study in collaboration with Kaiser Permanente (KP). The ethical approval of the study was primarily obtained from the Regional Committee for Research Ethics in Western Norway, with a waiver of informed consent because we used only aggregate data from the participating registries. Furthermore, ethical approval was obtained through the ethical approval process of each registry. This study followed the Strengthening the Reporting of Observational Studies in Epidemiology (STROBE) reporting guideline.

### Study Population

**Figure 1.**
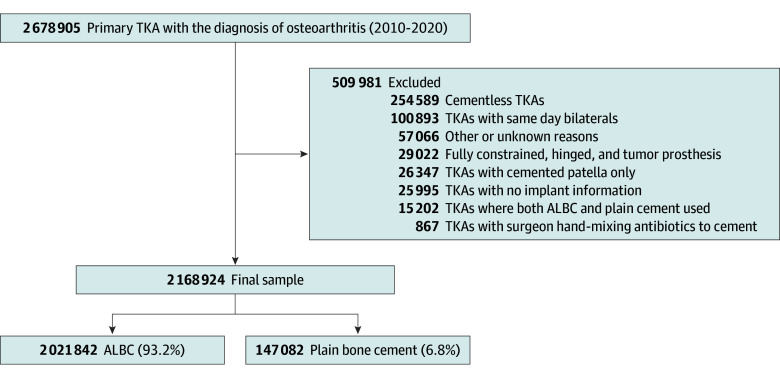
Cohort Selection Flowchart ALBC indicates antibiotic-loaded bone cement; TKA, total knee arthroplasty.

More than 2.1 million primary TKAs reported to 14 regional or national arthroplasty registries in Australia, Denmark, Finland, Germany, Italy, New Zealand, Norway, Romania, Sweden, Switzerland, the Netherlands, the UK, and the US from 2010 to 2020 were included (Figure 1). In 2022, all International Society of Arthroplasty Registries’ members were invited to participate in this study. Only 14 registries were able and had capacity (resources) to deliver the requested data. All but one of the participating registries reported high completeness of primary (>95%) and revision (≥85%) TKA.^20^

### Inclusion and Exclusion Criteria

To ensure a homogeneous study cohort, inclusion was restricted to all-cemented or hybrid (cemented tibia or femur) primary TKA in patients with osteoarthritis. Detailed inclusion and exclusion criteria are presented in Figure 1.

### Outcome Variables

The primary outcome was the 1-year revision rate for PJI, since a 2022 study from the US reported that limiting surveillance to 3 months misses more than 40% of infections in the first year after TKA.^33^ Secondary outcomes were revision for PJI or all causes at 3 months and 1, 5, and 10 years. Revision was defined as a reoperation with removal, addition, or exchange of part of a prosthesis or the whole prosthesis following the primary TKA. The participating registries used a standardized hierarchical list of diagnoses for revision TKA^34^ when reporting revisions.

### Follow-Up

Included TKAs were followed-up until the first revision or until December 31, 2021. Follow-up was censored at a time of patient death, migration out of the country or region (for regional registries), or health care membership termination (for KP).

### Data Extraction

A distributed data network not requiring centralized data storage was used.^35,36,37^ The data collection has been described in detail previously.^20^ This study combined aggregated data from each participating registry. The aggregation was performed in 2 stages. The NAR was the coordinating center.

The NAR, in collaboration with KP, created a data-sharing template, as well as a model template for Cox regressions, and distributed the templates to each participating registry for extraction of aggregate information of specifically defined data elements. First, each registry reported back to the NAR using the data sharing template summary statistics on patient and surgical characteristics according to type of cement used (ALBC vs plain bone cement), cause and number (and percentage) of revision surgeries, and estimated cumulative revision rate.^20^ Then, each registry evaluated and reported back the estimated rate and risk of revision for PJI and all causes following primary TKA using Cox regression analyses, reporting hazard rate ratios (HRR), β coefficients, SEs, and 95% CIs.^38,39^

### Statistical Analysis

Descriptive statistics, including frequencies, and percentages were used to describe each registry’s study sample. Descriptive statistics were conducted in Excel (Microsoft). *P* values were 2-sided, and statistical significance was set at *P* < .05. Data were analyzed from April to September 2023.

#### Individual Registry Analysis

Each registry calculated cumulative percentage revision (rate), calculated as 1 − Kaplan-Meier estimator of survivorship. Cox regression analysis was used to estimate HRRs of revision for PJI and all causes at 3 months and 1, 5, and 10 years, comparing plain bone cement with ALBC (risk). Each register calculated HRRs with 95% CIs for risk of revision in 3 Cox regression models: unadjusted Cox regression (model 1); Cox regression adjusted for age, sex, and surgery time period (model 2); and full Cox regression adjusted for variables the register had available, including age, sex, surgery time period, American Society of Anesthesiologists class, body mass index, patella resurfacing, fixation, stability, bearing mobility, and systemic antibiotic prophylactic administered (model 3). ALBC was the reference group in all regression models. Missing values were categorized as unknown for the Cox regression analysis. Findings from model 3 were used as the basis for the presentation in the results and throughout the discussion of this study. Only registries with both ALBC and plain bone cement used in more than 100 primary TKAs reported results from Cox regression analysis (excluding the Finnish; Swedish; and Bolzano, Italy, registries). In Norway, ALBC was used in 100% of primary TKAs. Hence, only 10 of 14 participating registries were included for the meta-analysis.

#### Meta-Analysis

Each registry’s estimate of the log HR (the β coefficients) with SEs from the Cox regression analysis was used to conduct advanced harmonized meta-analysis. Resulting HRRs and 95% CIs are presented in forest plots. A random-effects model (treating registries as a set of random effects) assuming some level of heterogeneity among data from individual registries^40^ was used, despite having less restricted inferences than the fixed-effects model.^41^ As the proportion of ALBC vs plain bone cement use in primary TKA varied among participating registries,^20^ we performed a sensitivity analysis to determine the influence of individual registries on the meta-analysis results.^40,42^ Stata software version 17 (StataCorp) was used for the meta-analyses.

## Results

This study included 2 168 924 primary TKAs, with 2 021 842 TKAs (93.2%) performed with ALBC (Table 1). Most TKAs were performed in female patients (1 290 940 TKAs [59.5%]) and patients aged 65 to 74 years (864 569 TKAs [39.9%]), fully cemented (1 999 556 TKAs [92.2%]), and in the period 2015-2020 (1 355 404 TKAs [62.5%]) (Table 2). The use of ALBC among participating registries varied from 34% in KP (US) to 100% in NAR (Norway) (Table 1).

**Table 1.** Primary and Revision TKA With ALBC vs Plain Bone Cement per Registry

Register (country)	Primary TKAs, No. (% of register)		No. (% of primary)			
			Revision TKAs for PJI		Revision TKAs for all causes	
	ALBC	Plain cement	ALBC	Plain cement	ALBC	Plain cement
Total	2 021 842 (93.2)	147 082 (6.8)	16 040 (0.8)	1454 (1.0)	58 339 (2.9)	4220 (2.9)
AOANJRR (Australia)	374 563 (96.3)	14 532 (3.7)	3737 (1.0)	149 (1.0)	11 848 (3.2)	533 (3.7)
DKR (Denmark)	37 442 (75.8)	11 935 (24.2)	390 (1.0)	115 (1.0)	1472 (3.9)	448 (3.8)
EPRD (Germany)	139 673 (98.4)	2263 (1.6)	1379 (1.0)	28 (1.2)	5078 (3.6)	88 (3.9)
FAR (Finland)	83 374 (99.9)	74 (0.1)	783 (0.9)	0	2713 (3.3)	0
KP (US)	42 005 (34.1)	81 072 (65.9)	439 (1.0)	815 (1.0)	1001 (2.4)	1971 (2.4)
LROI (the Netherlands)	195 155 (98.2)	3609 (1.8)	1475 (0.8)	28 (0.8)	7782 (4.0)	154 (4.3)
NAR (Norway)	40 709 (100)	0	474 (1.2)	0	1620 (4.0)	0
NJR (UK)	810 644 (99.4)	5124 (0.6)	4714 (0.6)	68 (1.3)	17 507 (2.2)	211 (4.1)
NZJR (New Zealand)	60 173 (81.6)	13 571 (18.4)	564 (0.9)	139 (1.0)	1640 (2.7)	435 (3.2)
PABZ (Italy)	4540 (99.9)	4 (0.1)	38 (0.8)	0	141 (3.1)	0
PATN (Italy)	970 (84.3)	180 (15.7)	9 (0.9)	0	17 (1.8)	3 (1.7)
RAR (Romania)	17 818 (57.8)	12 998 (42.2)	72 (0.4)	102 (0.8)	242 (1.4)	257 (2.0)
SAR (Sweden)	122 992 (>99.9)	41 (<0.1)	1323 (1.1)	1 (2.4)	3555 (2.9)	1 (2.4)
SIRIS (Switzerland)	91 784 (98.2)	1679 (1.8)	643 (0.7)	9 (<0.1)	3723 (4.1)	120 (7.1)

Abbreviations: ALBC, antibiotic-loaded bone cement; AOANJRR, Australian Orthopaedic Association National Joint Replacement Registry; DKR, Danish Knee Arthroplasty Registry; EPRD, German Arthroplasty Registry; FAR, Finnish Arthroplasty Register; KP, Kaiser Permanente Total Joint Replacement Registry; LROI, Dutch Arthroplasty Register; NAR, Norwegian Arthroplasty Register; NJR, National Joint Registry; NZJR, New Zealand Joint Registry; PABZ, Bolzano provincial register of knee prostheses (Autonomous Province of Bolzano, Italy); PATN, Trento provincial register of knee prostheses- (Autonomous Province of Trento, Italy); PJI, periprosthetic joint infection; RAR, Romanian Arthroplasty Register; SAR, Swedish Arthroplasty Register; SIRIS, Swiss National Implant Register; TKA, total knee arthroplasty.

**Table 2.** Demographic and Surgical Characteristics for Primary TKA With ALBC vs Plain Bone Cement (Pooled Data)

Characteristic	TKAs, No. (%)		
	ALBC	Plain cement	Total
Total No.	2 021 842 (93.2)	147 082 (6.8)	2 168 924 (100)
Age group, y^a^			
<55	124 982 (6.2)	8991 (6.1)	1133 973 (6.2)
55-64	481 415(23.8)	39 662 (27.0)	521 077 (24.0)
65-74	803 552 (39.7)	61 017 (41.5)	864 569 (39.9)
≥75	611 666 (30.3)	37 410 (25.4)	649 076 (29.9)
Missing or unknown	227 (<0.1)	2 (<0.1)	229 (<0.1)
Sex			
Male	822 170 (40.7)	55 814 (37.9)	877 984 (40.5)
Female	1 199 672 (59.3)	91 268 (62.1)	1 290 940 (59.5)
Operative side			
Right	1 066 396 (52.7)	76 373 (51.9)	1 142 769 (52.7)
Left	955 446 (47.3)	70 709 (48.1)	1 026 155 (47.3)
Patella resurfacing			
Yes	784 321 (38.8)	112 299 (76.4)	896 603 (41.3)
No	1 236 862 (61.2)	34 760 (23.6)	1 271 622 (58.6)
Missing or unknown	659 (<0.1)	23 (<0.1)	682 (<0.1)
Time period			
2010-2014	744 929 (38.8)	65 591 (46.6)	813 520 (37.5)
2015-2020	1 276 913 (63.2)	78 491 (53.4)	1 355 404 (62.5)
Fixation			
Both or all cemented	1 866 465 (92.3)	133 091 (90.5)	1 999 556 (92.2)
Hybrid (tibial cemented)	148 868 (7.4)	12 182 (8.3)	161 050 (7.4)
Reverse hybrid (tibial cementless)	5049 (0.2)	635 (0.4)	5688 (0.3)
Missing or unknown	1460 (0.1)	1170 (0.8)	2630 (0.1)
ASA classification^b^			
I	154 613 (7.9)	4419 (3.6)	159 032 (7.6)
II	1 132 095 (57.6)	70 988 (58.1)	1 203 083 (57.6)
III	398 545 (20.3)	35 800 (29.3)	434 345 (20.8)
≥IV	7309 (0.4)	809 (0.7)	8118 (0.4)
Missing or unknown	274 020 (13.9)	10 133 (8.3)	284 153 (13.6)
BMI^c^			
<18.5	2516 (0.1)	159 (0.1)	2675 (0.1)
18.5-24.9	173 804 (8.9)	14 388 (10.7)	188 192 (9.1)
25.0-29.9	499 121 (25.5)	37 662 (28.1)	536 783 (25.9)
30.0-34.9	436 413 (22.3)	34 068 (25.4)	470 481 (22.7)
35.0-39.9	213 315 (10.9)	18 135(13.5)	231 450 (112)
≥40.0	96 626 (4.9)	6762 (5.1)	103 388 (5.0)
Missing or unknown	536 010 (27.4)	22 726 (17.0)	536 010 (25.9)
Bearing mobility^d^			
Mobile	164 516 (9.0)	6751 (5.6)	171 267 (8.8)
Fixed	1 509 332 (82.2)	110 189 (90.8)	1 619 521 (82.8)
Missing or unknown	161 495 (8.8)	4378 (3.6)	165 873 (8.5)
Stability^e^			
Posterior	400 090 (20.7)	80 390 (54.8)	480 480 (23.1)
Minimally stabilized	1 284 841 (66.3)	61 479 (41.9)	1 346 320 (64.6)
Missing or unknown	252 567 (13.0)	4959 (3.4)	257 526 (12.4)
Systemic antibiotic prophylaxis used^f^	374 309 (99.5)	118 503 (99.0)	492 812 (99.4)

Abbreviations: ALBC, antibiotic-loaded bone cement; ASA, American Society of Anesthesiologists. BMI, body mass index (calculated as weight in kilograms divided by height in meters squared); TKA, total knee arthroplasty.

^a^ The Dutch Arthroplasty Register (n = 163) and Swiss National Implant Register (n = 66) lacked information on age.

^b^ Only 12 registries recorded information on ASA; the Danish Knee Arthroplasty Registry and Romanian Arthroplasty Register do not collect this information.

^c^ Only 10 registries recorded information on BMI; Norwegian Arthroplasty Register, Bolzano provincial register of knee prostheses (Autonomous Province of Bolzano, Italy), Trento provincial register of knee prostheses (Autonomous Province of Trento, Italy), and Romanian Arthroplasty Register do not collect this information.

^d^ Only 9 registries recorded information on bearing mobility; Danish Knee Arthroplasty Registry, German Arthroplasty Registry, New Zealand Joint Registry, Bolzano provincial register of knee prostheses (Autonomous Province of Bolzano, Italy), and Trento provincial register of knee prostheses (Autonomous Province of Trento, Italy) do not collect this information.

^e^ Only 12 registries recorded information on stability; Finnish Arthroplasty Register and Trento provincial register of knee prostheses (Autonomous Province of Trento, Italy) do not collect this information.

^f^ Only 8 registries (Danish Knee Arthroplasty Registry, Finnish Arthroplasty Register, Kaiser Permanente Total Joint Replacement Registry, Norwegian Arthroplasty Register, New Zealand Joint Registry, Bolzano provincial register of knee prostheses (Autonomous Province of Bolzano, Italy), Romanian Arthroplasty Register, and Swedish Arthroplasty Register) recorded information on systemic antibiotic prophylaxis.

### Crude Incidence of Revision for PJI and All Causes

Overall, 16 040 TKAs (0.8%) with ALBC and 1443 TKAs (1.0%) with plain bone cement were revised for PJI. Furthermore, 56 754 TKAs (2.8%) with ALBC and 4222 TKAs (2.9%) with plain bone cement were revised due to all causes (Table 1).

The cumulative 1-year revision rates following primary TKAs with ALBC and plain bone cement for each participating registry are presented in eFigure 1 and eFigure 2 in Supplement 1. Each registry reported a cumulative revision rate of less than 1% for PJI following primary TKA with ALBC (ranging from 0.21% in the UK to 0.80% in Denmark) and with plain bone cement (ranging from 0.23% in the Netherlands to 0.70% in Germany) (eFigure 1 in Supplement 1). Except for the German and Swiss registries, participating registries reported cumulative 1-year revision rates for all causes of 2% or less both for TKA with ALBC and with plain bone cement (eFigure 2 in Supplement 1).

### Results of Distributed Meta-Analyses

**Figure 2.**
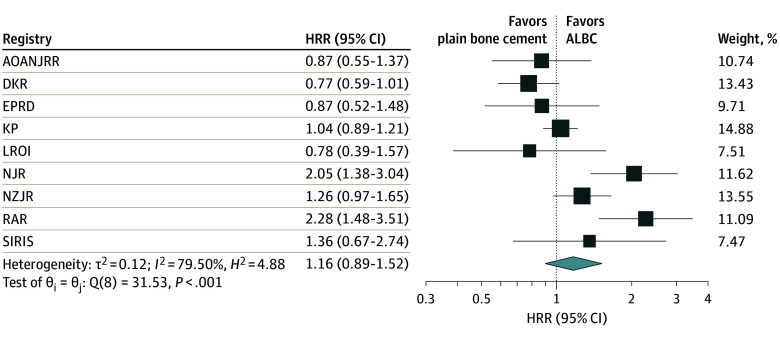
Meta-Analysis on Risk of Revision for Periprosthetic Joint Infection Following Primary Total Knee Arthroplasty With Antibiotic-Loaded Bone Cement (ALBC) vs Plain Bone Cement at 1 Year The meta-analysis was based on result from Cox regression analysis adjusted for age, sex, year of surgery, and all other variables available in each participating registry. The size of the square corresponds to the weight of each registry based on the number of total knee arthroplasties with plain bone cement in the registry. AOANJRR indicates Australian Orthopaedic Association National Joint Replacement Registry; DKR, Danish Knee Arthroplasty Registry; EPRD, German Arthroplasty Registry; KP, Kaiser Permanente Total Joint Replacement Registry; LROI, Dutch Arthroplasty Register; NJR, National Joint Registry; NZJR, New Zealand Joint Registry; RAR, Romanian Arthroplasty Register; SIRIS, Swiss National Implant Register.

**Figure 3.**
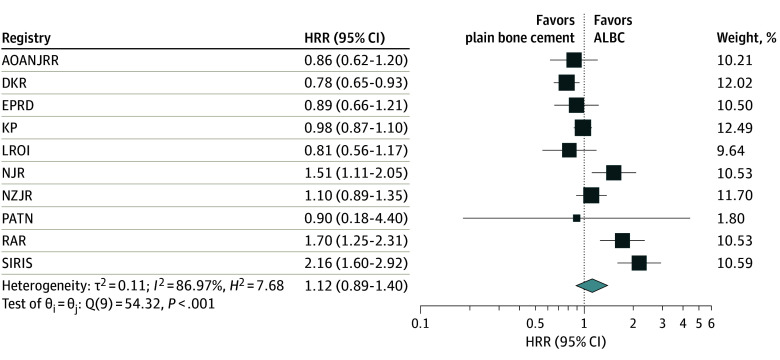
Meta-Analysis on Risk of Revision for All Causes Following Primary Total Knee Arthroplasty With Antibiotic-Loaded Bone Cement (ALBC) vs Plain Bone Cement at 1 Year The meta-analysis was based on result from Cox regression analysis adjusted for age, sex, year of surgery, and all other variables available in each participating registry. The size of the square corresponds to the weight of each registry based on the number of total knee arthroplasties with plain bone cement in the registry. AOANJRR indicates Australian Orthopaedic Association National Joint Replacement Registry; DKR, Danish Knee Arthroplasty Registry; EPRD, German Arthroplasty Registry; KP, Kaiser Permanente Total Joint Replacement Registry; LROI, Dutch Arthroplasty Register; NJR, National Joint Registry; NZJR, New Zealand Joint Registry; PATN, Trento provincial register of knee prostheses (Autonomous Province of Trento, Italy); RAR, Romanian Arthroplasty Register; SIRIS, Swiss National Implant Register.

Of 14 participating registries, 10 registries with 1 917 190 TKAs had sufficient numbers of TKAs in both study groups. Individual Cox regression analyses results from participating registries are reported in eTable 1 and eTable 2 in Supplement 1. The meta-analyses based on Cox regression found that the difference in risk of revision for PJI between primary TKA with ALBC compared with plain bone cement was not statistically significant (Figure 2; eFigures 3-5 in Supplement 1). For instance, the adjusted meta-analysis found that primary TKA with plain bone cement had no difference in risk of revision for PJI compared with TKA with ALBC at 1 year (HRR, 1.16; 95% CI, 0.89-1.52) (Figure 2) or at 3 months (HRR, 1.16; 95% CI, 0.75–1.79), 5 years (HRR, 1.17; 95% CI, 0.98-1.40), or 10 years (HRR, 1.17; 95% CI, 0.98-1.40) (eFigure 5 in Supplement 1). Similarly, we observed no significant differences for risk of all-cause revision following primary TKA with plain bone cement vs ALBC at 1 year (HRR, 1.12; 95% CI, 0.89-1.40) or at 3 months, 5 years, or 10 years (eFigures 6-8 in Supplement 1). However, substantial heterogeneity (*I*^2^ ≥ 75%; *P* = .001) was observed in the meta-analyses in all 3 Cox models (Figure 2 and Figure 3; eFigures 4-8 in Supplement 1).

### Sensitivity Analysis

The sensitivity analysis demonstrated the results of the meta-analysis for risk of revision for PJI were consistent as individual registries were stepwise removed from the meta-analysis (eTable 3 in Supplement 1). Findings were similar for risk of revision for all causes (eTable 4 in Supplement 1).

## Discussion

To our knowledge, this cohort study has the largest international registry-based meta-analysis performed to date comparing the association of ALBC vs plain bone cement with risk of revision for PJI and all causes following primary TKA. The cumulative 1-year revision rate for PJI ranged from 0.21% to 0.80% with ALBC and from 0.23% to 0.70% with plain bone cement. The meta-analysis revealed no significant differences in risk of revision for PJI or all causes following primary TKAs with ALBC vs plain bone cement.

Existing literature on the effectiveness of ALBC in primary TKA is controversial. In some studies, ALBC has been found to reduce the risk of revision for PJI,^43,44,45,46,47,48,49^ whereas other studies have reported no differences between ALBC and plain bone cement.^24,50,51,52,53,54,55,56,57,58,59,60,61,62,63^ One large randomized clinical trial comparing ALBC and plain bone cement with nearly 3000 TKAs also showed no difference in PJI; however, the antibiotics used were colistin and erythromycin.^53^ Some earlier individual registry–based studies have reported lower risk or similar revision rates for aseptic loosening in TKAs with ALBC compared with TKAs with plain bone cement.^16,19,49,52^ A registry-based study from the UK reported a lower risk of revision for all causes with use of ALBC compared with plain bone cement.^49^ Other studies even reported a higher risk of revision for infection in the ALBC group.^54,60,64^ These differences may be attributed to the study size, quality, variation in ALBC utilization, different settings, and differences in patient- and surgery-related characteristics. Our results have high external validity due to the large cohort size to detect small differences in event rates, addressing variation in ALBC utilization and antibiotics in the bone cement, and inclusion of different settings (single country vs international data). In our study, only cemented (fully or hybrid) primary TKAs due to osteoarthritis were included.

The strength of this study is that, to our knowledge, it is the first and largest international multiregister-based meta-analysis on risk of revision following TKA using ALBC compared with plain bone cement. Incorporating 14 registries across 3 continents provided the opportunity to examine rates of revision following primary TKA among participating countries and to increase the generalizability of the findings.

### Implications and Clinical Relevance

We found no evidence of an association of ALBC with reduced risk of revision for PJI compared with plain bone cement across the registries meta-analyzed. Thus, if we assume a noninferiority margin of 0.16 between the ALBC and the plain bone cement group, 625 patients need to undergo primary TKA surgery with plain bone cement to cause 1 extra revision for PJI compared with ALBC if the relative risk of 1.16 was assumed statistically significant.

Furthermore, earlier studies reported that routine use of ALBC in primary joint arthroplasty is not cost effective.^24,59,62,65^ Namba et al^19^ reported the cost differential between the 2 cement types as an extra $308 for 2 bags of ALBC compared with plain bone cement. In the US, for instance, with 790 000 TKAs yearly and 30% use of ALBC, this could equate to a savings of more than $72 million annually.

### Limitations

This study has some limitations. First, the global representativeness of the participating registries is limited, given the overrepresentation of registries from Europe and no or underrepresentation of registries from Africa, Asia, Latin America, and North America. Nevertheless, data from the participating registries provide important and relevant information on risk of revision following primary TKAs using either ALBC or plain bone cement.

Second, the data rely on accurate coding of implant information and are subject to reporting error. Most participating registries reported high completeness (>95%) of primary TKA, which shows that they undergo a rigorous process of internal auditing to ensure the accuracy of the collected data.^66,67,68^ Besides, the revision diagnosis is reported immediately after revision surgery and not after results from bacterial culture reports several days later. This could cause erroneous reporting, which might remain uncorrected in the register. A recent register study on revision hip arthroplasty found high accuracy (87%) of surgeon-reported revisions for PJI.^69^

Third, the inherent nature of registry data collection may rely on time of surgery, resulting in some inaccuracies in stated causes for revision. For example, revisions attributed to aseptic loosening may ultimately be driven by low-grade infection; thus, registries are likely to underreport infection as a cause of revision.^68^ However, it is very unlikely this underreporting is associated with a systematic bias between the groups in this study.

Fourth, this study included data from different national registries, potentially with different baseline characteristics of patients, surgical techniques, and perioperative protocols that inherently make it difficult to account for all possible confounding variables. Furthermore, the 10 registries included in the meta-analyses had high heterogeneity (*I*^2^ ≥ 75%), and heterogeneity diminishes the certainty of the findings.^70^ However, in this study, we used the random-effects models for the meta-analysis, considering that the number of procedures each participating registry contributes has a minor influence on the findings, diminishing potential inequality from the larger volume registries.^41,70^ In addition, we assessed the meta-analysis results with sensitivity analysis of the individual registries^70^ and found no change in estimates. Thus, we believe that the heterogeneity of the participating registries should not diminish the certainty of the findings.

Fifth, informative censoring (eg, time to patient death) for time to revision of the TKA may alter the observed risks for TKA revision. This could possibly be adjusted for using some sort of weighting.^71^ Furthermore, when investigating PJI, other types of TKA revisions would be plausible competing risks, which could be controlled for. Due to the nature of the data collection, with analyses performed separately at each register, including all aspects of possible competing risks and informative censoring would increase the complexity of the analytic scheme vastly. However, we do not believe that extending the analyses to competing risks and censoring would alter the overall findings of this study.

Sixth, we do not know what determines the choice of either type of cement for the individual patient, particularly in registries using both ALBC and plain bone cement. It could be the surgeon or department uses plain bone cement or ALBC for all patients (no selection) or patients at higher risk for infection receive ALBC but patients with lower risk receive plain bone cement (selection). If the latter was the case, this would skew the results in favor of plain bone cement.

Seventh, only 10 of 14 participating registries were included in the meta-analyses. We do not know whether the results might have changed if Finland; Bolzano, Italy; Norway; and Sweden had been included in the meta-analyses.

Eighth, this study was able to assess the association of ALBC use with risk of revision for PJI and for all cause revision following primary TKA. However, the impact of the cement type (brand), viscosity, type, and dose or duration of systemic antibiotic prophylaxis used were not taken into account, although variation in these covariates was reported among the participating registries.^20^ Furthermore, various types of bone cement were used in the different countries. However, a 2023 international study based on 16 regional or national registries, including 14 of the registries in this study, reported use of high-viscosity (92%) and gentamicin-containing (94%) ALBC in primary TKAs.^20^ Thus, we believe that the heterogeneity among bone cement used should not alter the certainty of the findings.

## Conclusions

This cohort study found no difference in risk of revision for PJI or all causes for use of ALBC vs plain bone cement for primary TKA. Any additional costs of ALBC should be considered in the context of the overall health care delivery system and its relative value in reducing revision risk. However, given the substantial variation in cohort size, patient characteristics, and clinical practice across the registries that might lead to variations in risk of revision in PJI, these findings need to be interpreted with caution.

## Notes

Supplement 1. **eFigure 1.** Cumulative Revision Rates Due to PJI Following Primary TKA With ALBC vs Plain Bone Cement **eFigure 2.** Cumulative Revision Rates Due to All Causes Following Primary TKA With ALBC vs Plain Bone Cement **eFigure 3.** Meta-Analysis on Risk of Revision Due to PJI Following Primary TKA With ALBC vs Plain Bone Cement (Model 1) **eFigure 4.** Meta-Analysis on Risk of Revision Due to PJI Following Primary TKA With ALBC vs Plain Bone Cement (Model 2) **eFigure 5.** Meta-Analysis on Risk of Revision Due to PJI Following Primary TKA With ALBC vs Plain Bone Cement (Model 3) **eFigure 6.** Meta-Analysis on Risk of Revision Due to All Causes Following Primary TKA With ALBC vs Plain Bone Cement (Model 1) **eFigure 7.** Meta-Analysis on Risk of Revision Due to All Causes Following Primary TKA With ALBC vs Plain Bone Cement (Model 2) **eFigure 8.** Meta-Analysis on Risk of Revision Due to All Causes Following Primary TKA With ALBC vs Plain Bone Cement (Model 3) **eTable 1.** Cox Regression Results From Individual Registries of Revision for PJI Following Primary TKA With ALBC vs Plain Bone Cement **eTable 2.** Cox Regression Results From Individual Registries of Revision Due to All Causes Following Primary TKA With ALBC vs Plain Bone Cement **eTable 3.** Sensitivity Analysis of the Meta-Analyses (model 2) of Revision for PJI and All Causes to Determine How Sensitive the Meta-Analyses Results Are to the Results of Individual Registry Contributions **eTable 4.** Sensitivity Analysis of Meta-Analyses (model 3) of Revision for PJI and All Causes to Determine How Sensitive the Meta-Analyses Results Are to the Results of Individual Registry Contributions

Supplement 2.
Data Sharing Statement
