# Supplementary material for: Periprosthetic Joint Infection After Total Knee Arthroplasty With or Without Antibiotic Bone Cement
Source: JAMA Netw Open. 2024 May 23;7(5):e2412898. doi: 10.1001/jamanetworkopen.2024.12898 (PMC11117087; doi:10.1001/jamanetworkopen.2024.12898)
Supplement: Supplement 1. — eFigure 1. Cumulative Revision Rates Due to PJI Following Primary TKA With ALBC vs Plain Bone Cement eFigure 2. Cumulative Revision Rates Due to All Causes Following Primary TKA With ALBC vs Plain Bone Cement eFigure 3. Meta-Analysis on Risk of Revision Due to PJI Following Primary TKA With ALBC vs Plain Bone Cement (Model 1) eFigure 4. Meta-Analysis on Risk of Revision Due to PJI Following Primary TKA With ALBC vs Plain Bone Cement (Model 2) eFigure 5. Meta-Analysis on Risk of Revision Due to PJI Following Primary TKA With ALBC vs Plain Bone Cement (Model 3) eFigure 6. Meta-Analysis on Risk of Revision Due to All Causes Following Primary TKA With ALBC vs Plain Bone Cement (Model 1) eFigure 7. Meta-Analysis on Risk of Revision Due to All Causes Following Primary TKA With ALBC vs Plain Bone Cement (Model 2) eFigure 8. Meta-Analysis on Risk of Revision Due to All Causes Following Primary TKA With ALBC vs Plain Bone Cement (Model 3) eTable 1. Cox Regression Results From Individual Registries of Revision for PJI Following Primary TKA With ALBC vs Plain Bone Cement eTable 2. Cox Regression Results From Individual Registries of Revision Due to All Causes Following Primary TKA With ALBC vs Plain Bone Cement eTable 3. Sensitivity Analysis of the Meta-Analyses (model 2) of Revision for PJI and All Causes to Determine How Sensitive the Meta-Analyses Results Are to the Results of Individual Registry Contributions eTable 4. Sensitivity Analysis of Meta-Analyses (model 3) of Revision for PJI and All Causes to Determine How Sensitive the Meta-Analyses Results Are to the Results of Individual Registry Contributions [file jamanetwopen-e2412898-s001.pdf]

Supplemental Online Content

Leta TH, Lie SA, Fenstad AM, et al. Periprosthetic Joint Infection After Total Knee Arthroplasty With or Without Antibiotic Bone Cement. JAMA Netw Open. 2024;7(5):e2412898. doi:10.1001/jamanetworkopen.2024.12898

eFigure 1. Cumulative Revision Rates Due to PJI Following Primary TKA With ALBC vs Plain Bone Cement
eFigure 2. Cumulative Revision Rates Due to All Causes Following Primary TKA With ALBC vs Plain Bone Cement
eFigure 3. Meta-Analysis on Risk of Revision Due to PJI Following Primary TKA With ALBC vs Plain Bone Cement (Model 1)
eFigure 4. Meta-Analysis on Risk of Revision Due to PJI Following Primary TKA With ALBC vs Plain Bone Cement (Model 2)
eFigure 5. Meta-Analysis on Risk of Revision Due to PJI Following Primary TKA With ALBC vs Plain Bone Cement (Model 3)
eFigure 6. Meta-Analysis on Risk of Revision Due to All Causes Following Primary TKA With ALBC vs Plain Bone Cement (Model 1)
eFigure 7. Meta-Analysis on Risk of Revision Due to All Causes Following Primary TKA With ALBC vs Plain Bone Cement (Model 2)
eFigure 8. Meta-Analysis on Risk of Revision Due to All Causes Following Primary TKA With ALBC vs Plain Bone Cement (Model 3)
eTable 1. Cox Regression Results From Individual Registries of Revision for PJI Following Primary TKA With ALBC vs Plain Bone Cement
eTable 2. Cox Regression Results From Individual Registries of Revision Due to All Causes Following Primary TKA With ALBC vs Plain Bone Cement
eTable 3. Sensitivity Analysis of the Meta-Analyses (model 2) of Revision for PJI and All Causes to Determine How Sensitive the Meta-Analyses Results Are to the Results of Individual Registry Contributions
eTable 4. Sensitivity Analysis of Meta-Analyses (model 3) of Revision for PJI and All Causes to Determine How Sensitive the Meta-Analyses Results Are to the Results of Individual Registry Contributions

This supplemental material has been provided by the authors to give readers additional information about their work.

© 2024 Leta TH et al. *JAMA Network Open.*

eFigure 1: Cumulative percent revision (one minus Kaplan-Meier estimator) due to PJI following primary TKA with (a) ALBC vs (b) plain bone cement.

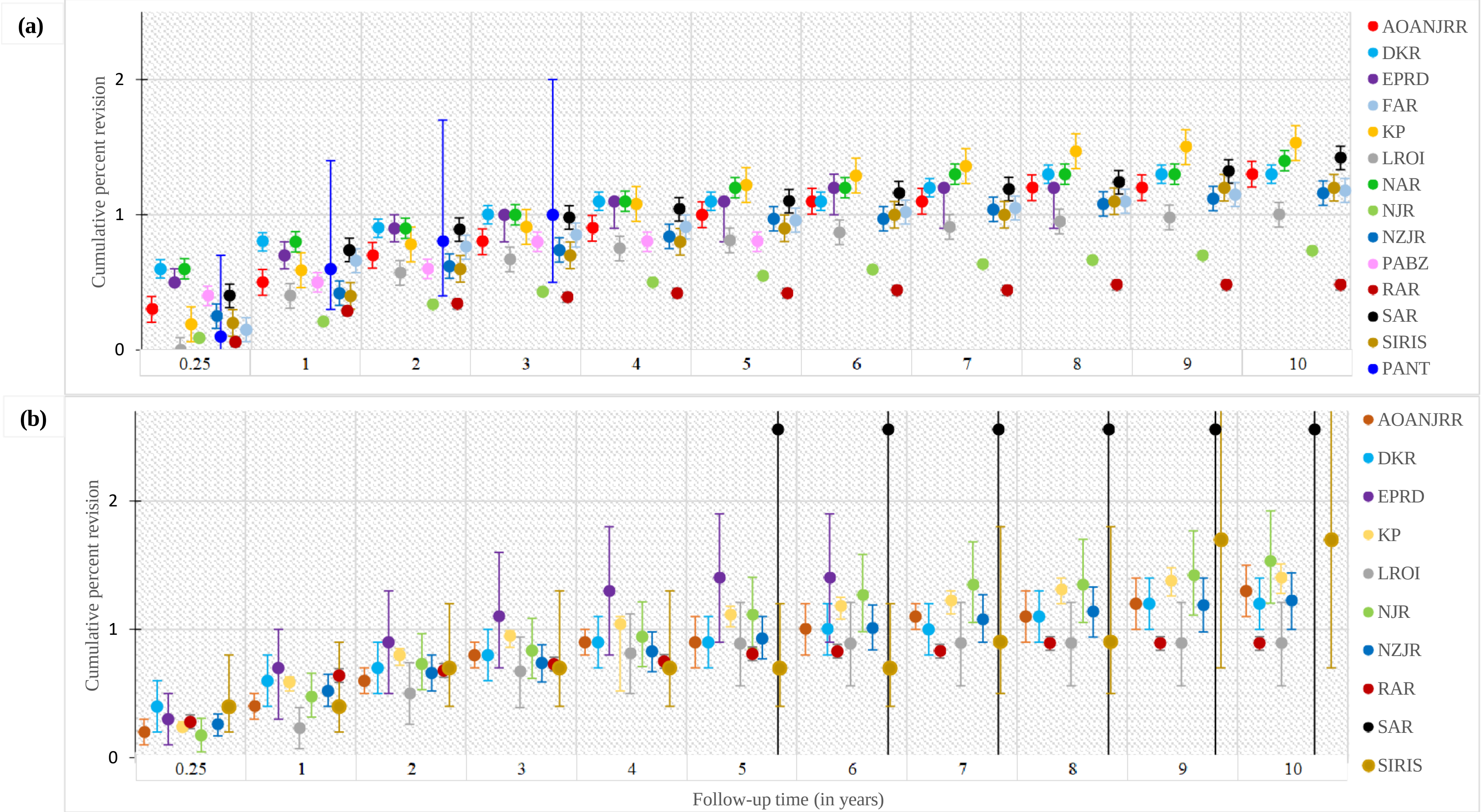

FAR, PABZ, and PATN had reported no revision due to infection following primary TKA with plain bone cement. NAR reported 100% use of ALBC in primary TKA.

\

© 2024 Leta TH et al. *JAMA Network Open.*

eFigure 2: Cumulative percent revision (one minus Kaplan-Meier estimator) due to all-causes following primary TKA with (a) ALBC vs (b) plain bone cement.

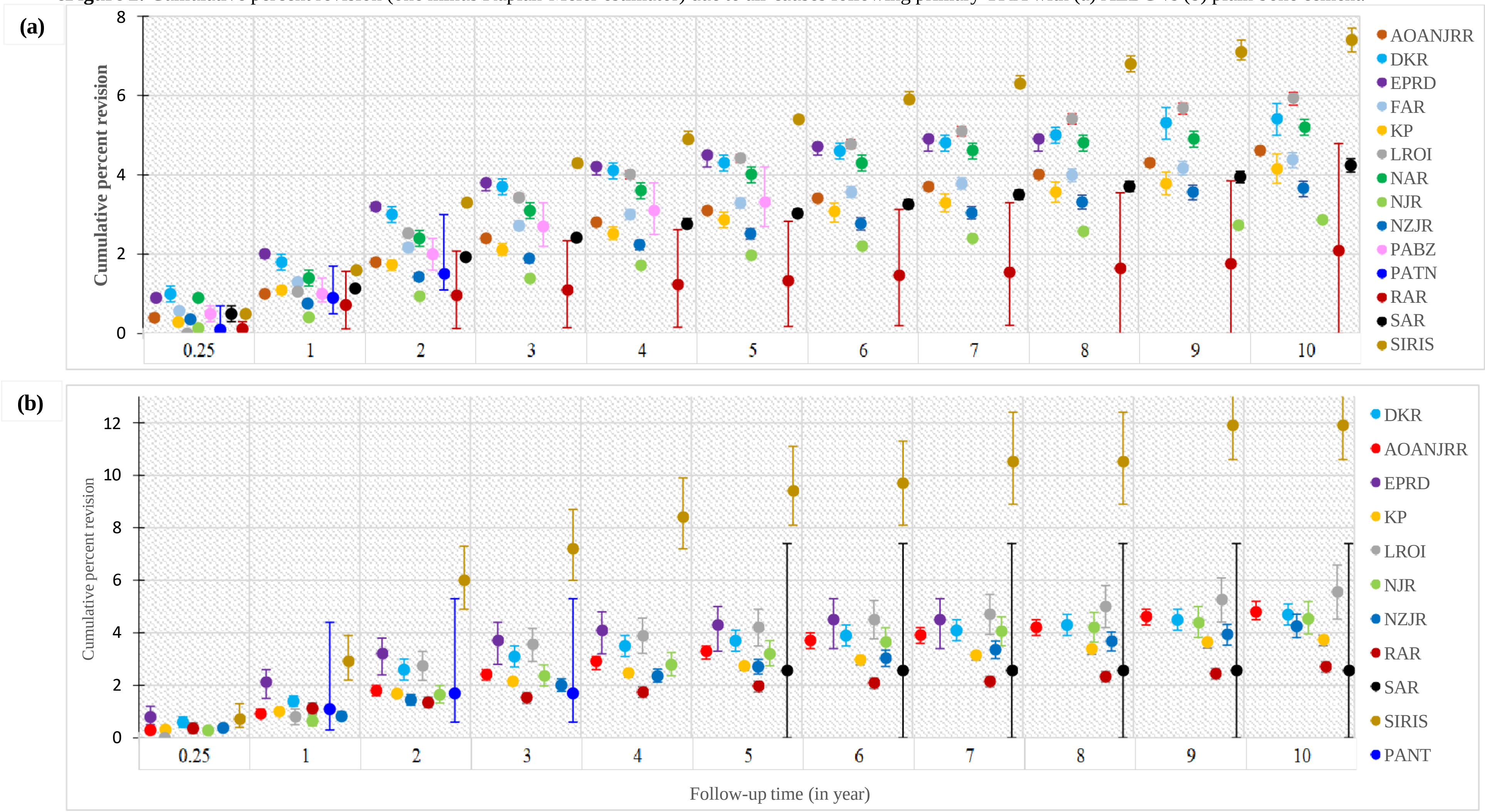

FAR & PABZ had reported no revision due to infection following primary TKAs with plain bone cement. NAR reported 100% use of ALBC in primary TKAs.

AOANJRR = The Australian Orthopaedic Association National Joint Replacement Registry
DKR = The Danish Knee Arthroplasty Registry
EPRD = The German Arthroplasty Registry

\

FAR	= The Finnish Arthroplasty Register
KP	= Kaiser Permanente Total Joint Replacement Registry
LROI	= Dutch Arthroplasty Register
NAR	= The Norwegian Arthroplasty Register
NJR	=The National Joint Registry
NZJR	= The New Zealand Joint Registry
PABZ	= The Bolzano provincial register of knee prostheses- (Autonomous Province of Bolzano-Italy)
PATN	= The Trento provincial register of knee prostheses- (Autonomous Province of Trento-Italy)
RAR	= Romanian Arthroplasty Register
SAR	= The Swedish Arthroplasty Register
SIRIS	= Swiss National Implant Register

© 2024 Leta TH et al. *JAMA Network Open.*

eFigure 3: Meta-analysis on risk of revision due to PJI following primary TKA with ALBC vs plain bone cement. The meta-analysis was based on results from unadjusted Cox-regression analysis.[a]

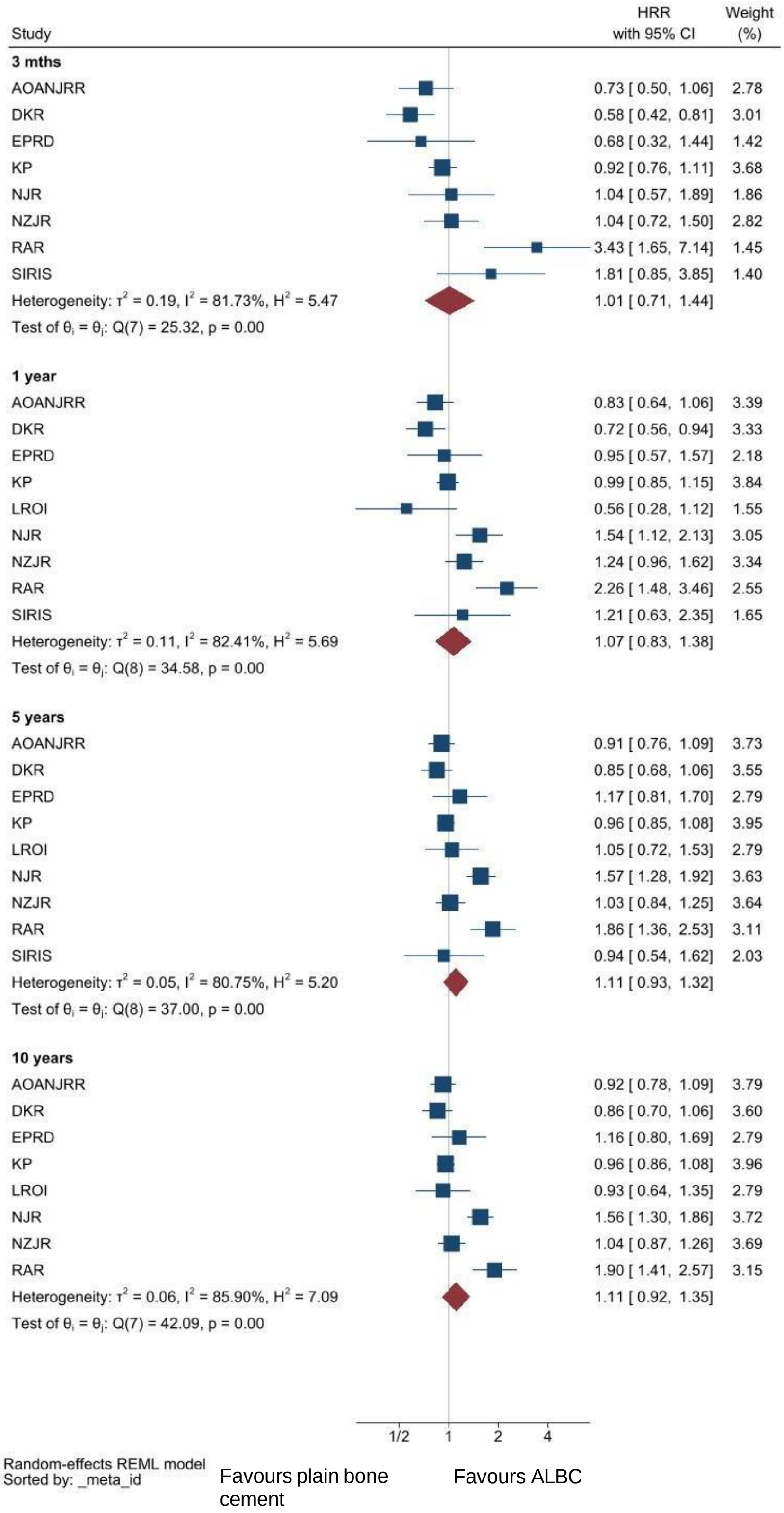

[a] The size of the square in the forest plot corresponds to each registries weighted based on the number of TKA with plain bone cement in the registry.

eFigure 4: Meta-analysis on risk of revision due to PJI following primary TKA with ALBC vs plain bone cement. The meta-analysis was based on the results from Cox-regression analysis adjusted for age, sex, year of surgery [time period].[a]

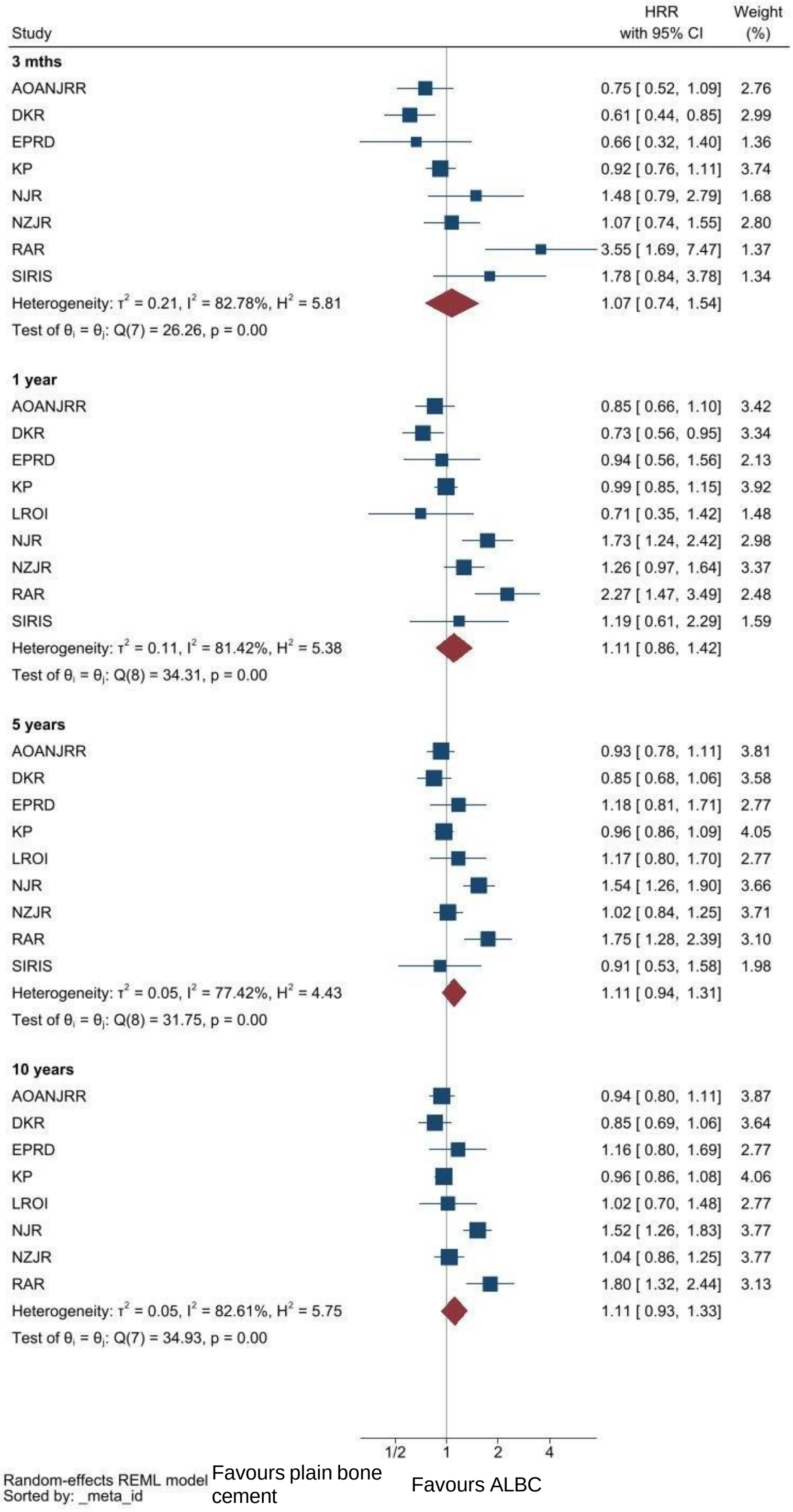

[a] The size of the square in the forest plot corresponds to each registries weighted based on the number of TKA with plain bone cement in the registry.

eFigure 5: Meta-analysis on risk of revision due to PJI following primary TKA with ALBC vs plain bone cement. The meta-analysis was based on result from Cox-regression analysis adjusted for age, sex, year of surgery [time period], and all other variables available in each participating registry.[a]

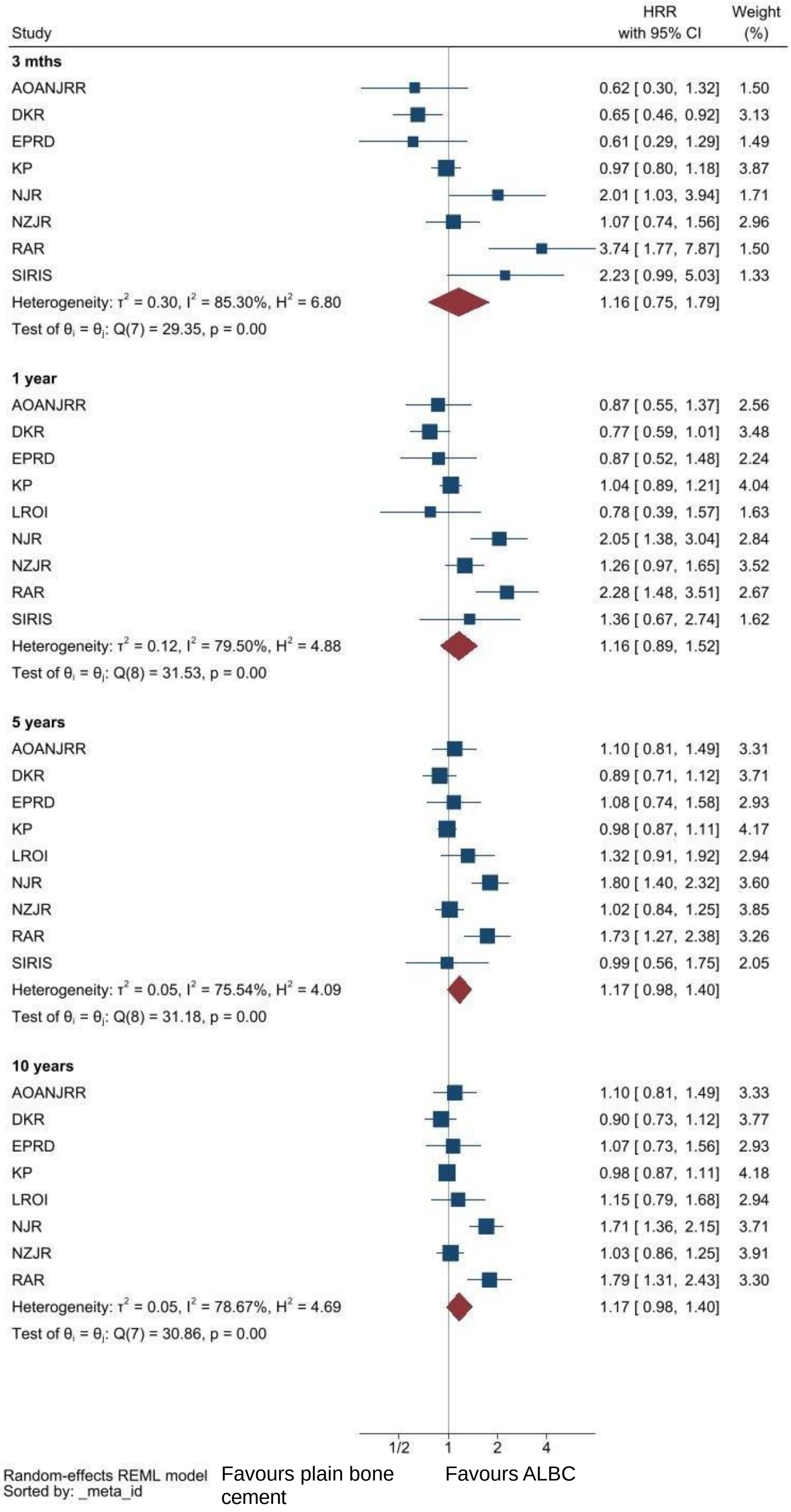

[a] The size of the square in the forest plot corresponds to each registry weighted based on the number of TKA with plain bone cement in the registry.

eFigure 6: Meta-analysis on risk of revision due to all-causes following primary TKA with ALBC vs plain bone cement. The meta-analysis was based on result from unadjusted Cox-regression analysis. [a]

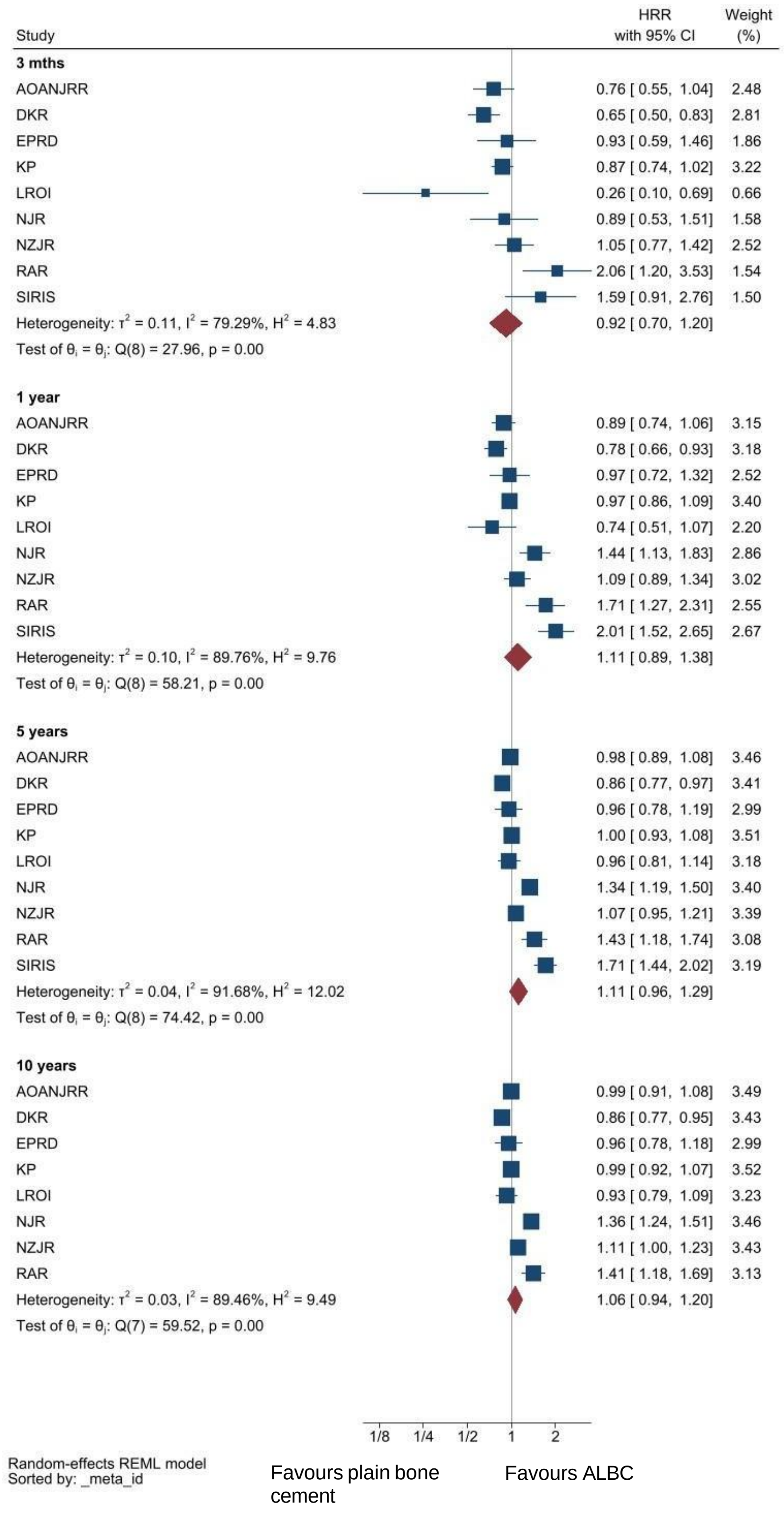

[a] The size of the square in the forest plot corresponds to each registry weighted based on the number of TKA with plain bone cement in the registry.

eFigure 7: Meta-analysis on risk of revision due to all-causes following primary TKA with ALBC vs plain bone cement. The meta-analysis was based on result from Cox-regression analysis adjusted for age, sex, and year of surgery [time period].[a]

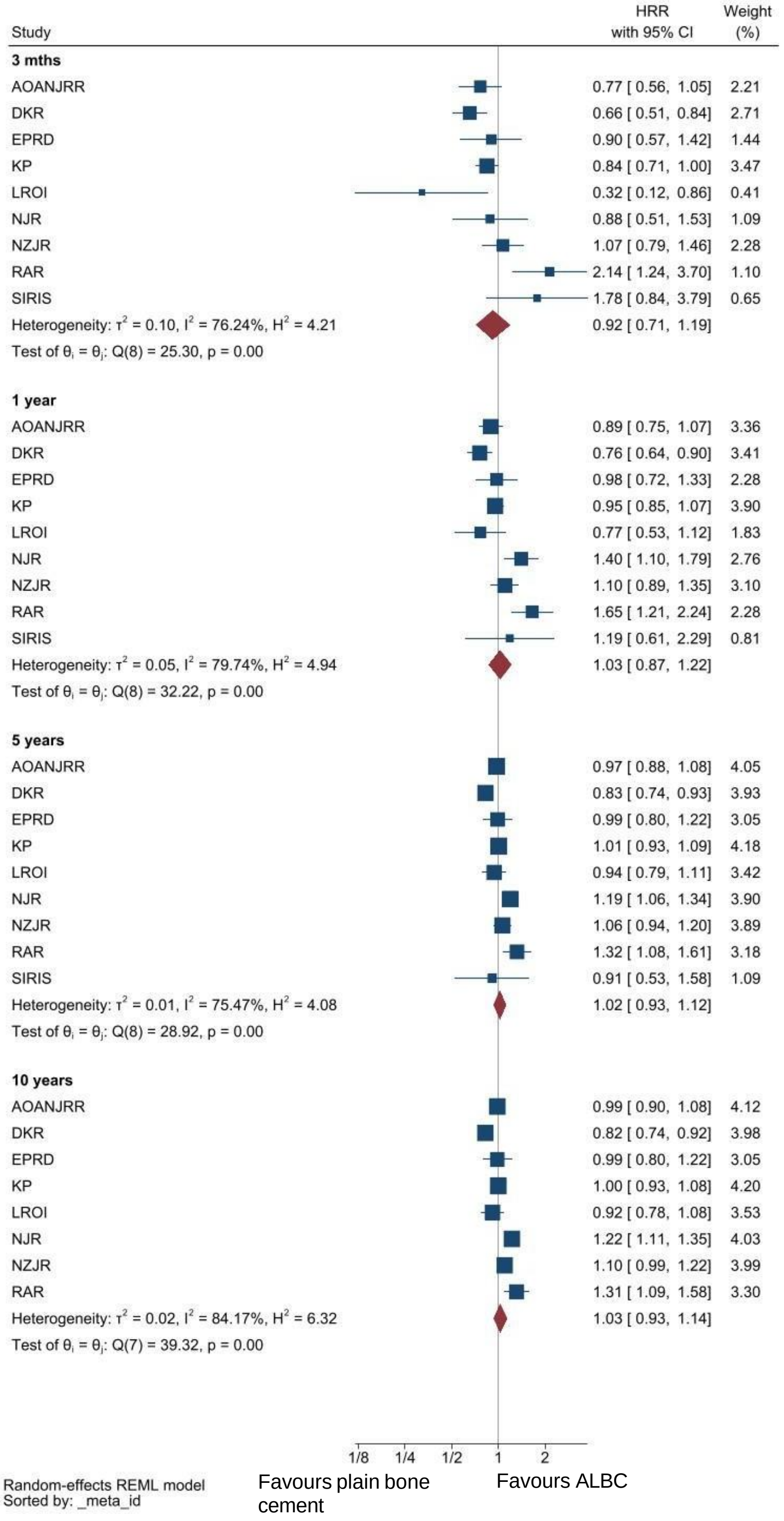

[a] The size of the square in the forest plot corresponds to each registry weighted based on the number of TKA with plain bone cement in the registry.

eFigure 8: Meta-analysis on risk of revision due to all-causes following primary TKA with ALBC vs plain bone cement. The meta-analysis was based on result from Cox-regression analysis adjusted for age, sex, year of surgery [time period], and all other variables available in each participating registry.[a]

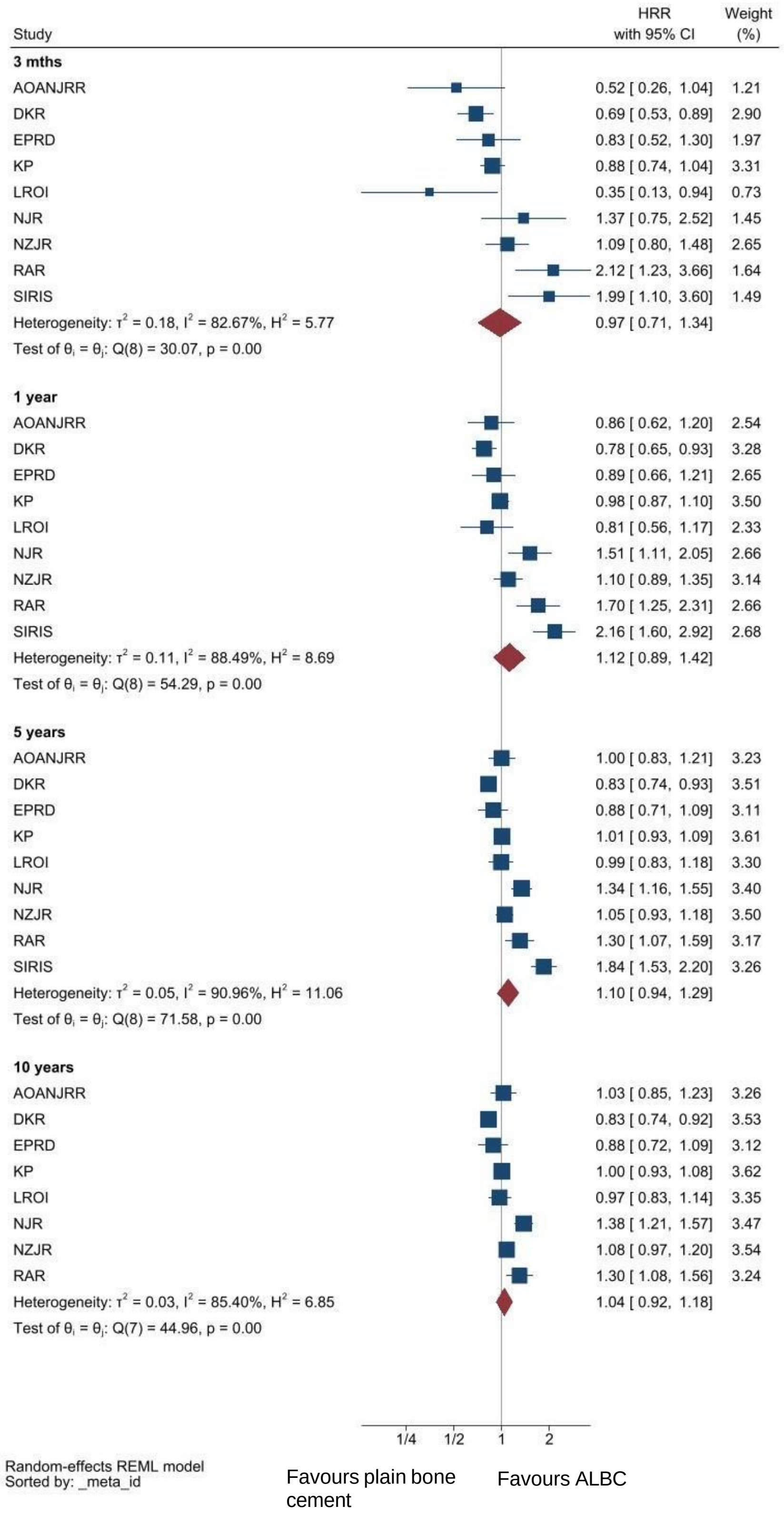

[a] The size of the square in the forest plot corresponds to each registry weighted based on the number of TKA with plain bone cement in the registry.

eTable 1: The Cox regression results from individual registries of revision for PJI following primary TKA with ALBC vs. plain bone cement (2010-2020).

	HR (95% CI)[a]			
**Register (country)[b]**	**3-months**	**1-year**	**5-years**	**10-years**
**AOANJRR (Australia) n=389,095**				
Number of revisions due to PJI	1,018	1,994	3,530	3,886
Cox-Model 1[c]	0.73 (0.50-1.06)	0.83 (0.64-1.06)	0.91 (0.76-1.09)	0.92 (0.78-1.09)
Cox-Model 2[d]	0.75 (0.52-1.10)	0.85 (0.66-1.10)	0.93 (0.78-1.11)	0.94 (0.80-1.11)
Cox-Model 3[e]	0.62 (0.30-1.32)	0.87 (0.55-1.37)	1.10 (0.81-1.49)	1.10 (0.81-1.49)
**DKR (Denmark) n=49,377**				
Number of revisions due to PJI	266	359	467	505
Cox-Model 1[c]	0.59 (0.42-0.81)	0.72 (0.56-0.94)	0.85 (0.68-1.09)	0.86 (0.70-1.06)
Cox-Model 2[d]	0.61 (0.44-0.85)	0.73 (0.56-0.95)	0.85 (0.68-1.06)	0.85 (0.69-1.05)
Cox-Model 3[e]	0.65 (0.46-0.92)	0.77 (0.59-1.01)	0.89 (0.71-1.12)	0.90 (0.73-1.12)
**EPRD (Germany) n=141,936**				
Number of revisions due to PJI	639	991	1,395	1,407
Cox-Model 1[c]	0.68 (0.32-1.44)	0.95 (0.57-1.57)	1.17 (0.81-1.70)	1.16 (0.80-1.69)
Cox-Model 2[d]	0.66 (0.32-1.40)	0.94 (0.56-1.56)	1.18 (0.81-1.71)	1.16 (0.80-1.69)
Cox-Model 3[e]	0.61 (0.29-1.29)	0.87 (0.52-1.46)	1.08 (0.74-1.58)	1.07 (0.73-1.56)
**KP (USA) n=123,418**				
Number of revisions due to PJI	439	705	1,168	1,254
Cox-Model 1[c]	0.85 (0.70-1.03)	0.90 (0.77-1.05)	0.91 (0.81-1.02)	0.91 (0.81-1.02)
Cox-Model 2[d]	0.86 (0.71-1.05)	0.92 (0.78-1.07)	0.92 (0.82-1.04)	0.92 (0.82-1.04)
Cox-Model 3[e]	0.91 (0.74-1.10)	0.96 (0.82-1.12)	0.94 (0.83-1.06)	0.94 (0.83-1.06)
**LROI (the Netherlands) n=198,712**				
Number of revisions due to PJI	521	778	1,392	1,500
Cox-Model 1[c]	0.0 (0.0-100)	0.56 (0.28-1.12)	1.05 (0.72-1.53)	0.93 (0.64-1.35)
Cox-Model 2[d]	0.0 (0.0-100)	0.71 (0.35-1.42)	1.17 (0.80-1.70)	1.02 (0.70-1.48)
Cox-Model 3[e]	0.0 (0.0-100)	0.78 (0.39-1.57)	1.32 (0.91-1.92)	1.15 (0.79-1.68)
**NJR (UK) n=815,698**				
Number of revisions	735	1,725	4,247	4,885
Cox-Model 1[c]	1.04 (0.57-1.90)	**1.55 (1.12-2.13)**	**1.57 (1.28-1.92)**	**1.56 (1.23-1.86)**
Cox-Model 2[d]	1.48 (0.79-2.79)	**1.73 (1.24-2.41)**	**1.55 (1.26-1.90)**	**1.52 (1.26-1.83)**
Cox-Model 3[e]	**2.02 (1.03-3.95)**	**2.05 (1.38-3.04)**	**1.80 (1.41-2.32)**	**1.71 (1.36-2.15)**
**NZJR (New Zealand) n=73,713**				
Number of revisions due to PJI	184	324	624	700
Cox-Model 1[c]	1.04 (0.72-1.50)	1.24 (0.96-1.62)	1.03 (0.84-1.25)	1.04 (0.87-1.26)
Cox-Model 2[d]	1.07 (0.74-1.55)	1.26 (0.97-1.64)	1.02 (0.84-1.25)	1.04 (0.86-1.25)
Cox-Model 3[e]	1.07 (0.74-1.56)	1.26 (0.97-1.65)	1.02 (0.84-1.25)	1.03 (0.86-1.25)
**PATN (Italy) n=1,150[f]**				
Number of revisions due to PJI	2	6		
Cox-Model 1[c]	0.00	0.00		
Cox-Model 2[d]	0.00	0.00		
Cox-Model 3[e]	0.00	0.00		
**RAR (Romania) n=30,813**				
Number of revisions due to PJI	35	90	165	174
Cox-Model 1[c]	**3.43 (1.65-7.14)**	**2.26 (1.48-3.47)**	**1.86 (1.36-2.53)**	**1.90 (1.41-2.57)**
Cox-Model 2[d]	**3.56 (1.69-7.47)**	**2.27 (1.47-3.49)**	**1.75 (1.28-2.39)**	**1.80 (1.32-2.44)**
Cox-Model 3[e]	**3.74 (1.78-7.86)**	**2.28 (1.48-3.51)**	**1.74 (1.27-2.38)**	**1.79 (1.32-2.43)**
**SIRIS (Switzerland) n=93,463**				
Number of revisions due to PJI	218	414	760	
Cox-Model 1[c]	1.81 (0.85-3.85)	1.21 (0.63-2.35)	0.93 (0.54-1.62)	
Cox-Model 2[d]	1.78 (0.84-3.79)	1.19 (0.61-2.29)	0.91 (0.53-1.58)	
Cox-Model 3[e]	2.23 (0.99-5.03)	1.36 (0.67-2.74)	0.99 (0.56-1.75)	

CI=confidence interval. HR=hazard ratio.

[a] ALCB was the reference group. Bold HR (95% CI) indicates significance with P-value <0.05.
[b] FAR, NAR, PABZ, and SAR were not evaluated in the meta-analysis as there were <100 TKA where plain bone cement was used.

c Cox-model 1 refers to unadjusted model.
d Cox-model 2 refers to model adjusted for age (<55, 55-64, **65-74**, >74), sex (**male**, female), and time period (**2010-2014**, 2015-2020).
e Cox-model 3 refers to fully adjusted model for age (<55, 55-64, **65-74**, >74), sex (**male**, female), time period (**2010-2014**, 2015-2020), ASA (**1**, 2, 3, ≥4), BMI (<18.5, **18.5-24.9**, 25.0-29.9, 30.0-34.9, 35.0-39.9, ≥40), patella resurfacing (**yes**/no), fixation (**fully cemented**, hybrid [cemented tibia], inverse hybrid [cemented femur]), systemic antibiotic prophylactic administered (**yes**, no, unknown), bearing mobility (**fixed**, mobile), and stability (**minimally stabilized**, posterior stabilized). Bold text indicates reference for covariates in the models.
[f] Plain bone cement was used only in 180 TKA and follow-up was less than 5 years.

AOANJRR = The Australian Orthopaedic Association National Joint Replacement Registry
DKR = The Danish Knee Arthroplasty Registry
EPRD = The German Arthroplasty Registry
FAR = The Finnish Arthroplasty Register
KP = Kaiser Permanente Total Joint Replacement Registry
LROI = Dutch Arthroplasty Register
NAR = The Norwegian Arthroplasty Register
NJR =The National Joint Registry
NZJR = The New Zealand Joint Registry
PABZ = The Bolzano provincial register of knee prostheses- (Autonomous Province of Bolzano-Italy)
PATN = The Trento provincial register of knee prostheses- (Autonomous Province of Trento-Italy)
RAR = Romanian Arthroplasty Register
SAR = The Swedish Arthroplasty Register
SIRIS = Swiss National Implant Register

eTable 2: The Cox regression results from individual registries of revision due to all-causes following primary TKA for ALBC vs. plain bone cement (2010-2020).

	HR (95% CI)[a]			
**Register (country)[b]**	**3-months**	**1-year**	**5-years**	**10-years**
**AOANJRR (Australia) n=389,095**				
Number of revisions	1,403	3,816	10,606	12,381
Cox-Model 1[c]	0.76 (0.55-1.04)	0.89 (0.74-1.06)	0.98 (0.89-1.08)	0.99 (0.91-1.08)
Cox-Model 2[d]	0.77 (0.56-1.05)	0.89 (0.75-1.07)	0.97 (0.88-1.08)	0.99 (0.90-1.07)
Cox-Model 3[e]	0.52 (0.26-1.04)	0.86 (0.62-1.20)	1.00 (0.83-1.21)	1.03 (0.86-1.23)
**DKR (Denmark) n=49,377**				
Number of revisions	437	823	1,751	1,913
Cox-Model 1[a]	0.65 (0.50-0.83)	0.78 (0.66-0.93)	0.86 (0.77-0.97)	0.86 (0.77-0.95)
Cox-Model 2[b]	0.66 (0.51-0.84)	0.76 (0.64-0.90)	0.83 (0.74-0.93)	0.83 (0.74-0.92)
Cox-Model 3[c]	0.69 (0.53-0.89)	0.78 (0.65-0.93)	0.83 (0.74-0.93)	0.83 (0.74-0.92)
**EPRD (Germany) n=141,936**				
Number of revisions	1,279	2,699	5,103	5,166
Cox-Model 1[c]	0.93 (0.59-1.46)	0.97 (0.72-1.32)	0.96 (0.78-1.19)	0.96 (0.78-1.18)
Cox-Model 2[d]	0.90 (0.57-1.42)	0.98 (0.72-1.33)	0.99 (0.80-1.22)	0.99 (0.80-1.22)
Cox-Model 3[e]	0.83 (0.52-1.31)	0.89 (0.66-1.21)	0.88 (0.71-1.10)	0.88 (0.71-1.09)
**KP (USA) n=123,077**				
Number of revisions	585	1,188	2,674	2,969
Cox-Model 1[c]	**0.84 (0.71-0.99)**	0.89 (0.79-1.00)	0.95 (0.88-1.03)	0.95 (0.88-1.02)
Cox-Model 2[d]	**0.84 (0.71-0.99)**	0.90 (0.80-1.01)	0.97 (0.90-1.05)	0.97 (0.90-1.04)
Cox-Model 3[e]	0.87 (0.73-1.04)	0.92 (0.81-1.03)	0.97 (0.89-1.05)	0.96 (0.89-1.04)
**LROI (the Netherlands) n=198,712**				
Number of revisions	836	2,054	7,114	7,902
Cox-Model 1[c]	0.26 (0.10-0.69)	0.74 (0.51-1.07)	0.96 (0.81-1.14)	0.93 (0.79-1.09)
Cox-Model 2[d]	0.32 (0.12-0.86)	0.77 (0.53-1.12)	0.94 (0.79-1.12)	0.91 (0.78-1.07)
Cox-Model 3[e]	0.35 (0.13-0.94)	0.81 (0.56-1.17)	0.99 (0.83-1.18)	0.97 (0.83-1.14)
**NJR (UK) n=815,698**				
Number of revisions	1,092	3,358	14,925	18,166
Cox-Model 1[c]	0.89 (0.53-1.51)	**1.44 (1.13-1.83)**	**1.34 (1.19-1.50)**	**1.37 (1.24-1.51)**
Cox-Model 2[d]	0.88 (0.65-1.97)	**1.41 (1.10-1.80)**	**1.19 (1.06-1.34)**	**1.22 (1.11-1.35)**
Cox-Model 3[e]	1.38 (0.75-2.52)	**1.51 (1.11-2.05)**	**1.34 (1.15-1.55)**	**1.38 (1.22-1.56)**
**NZJR (New Zealand) n=73,739**				
Number of revisions	267	571	1,709	2063
Cox-Model 1[c]	1.05 (0.77-1.42)	1.09 (0.89-1.34)	1.07 (0.95-1.21)	1.11 (0.99-1.23)
Cox-Model 2[d]	1.07 (0.79-1.46)	1.10 (0.89-1.35)	1.06 (0.94-1.20)	1.10 (0.99-1.22)
Cox-Model 3[e]	1.09 (0.80-1.48)	1.10 (0.89-1.35)	1.05 (0.93-1.18)	1.08 (0.97-1.20)
**PATN (Italy) n=1,150[f]**				
Number of revisions	2	11		
Cox-Model 1[c]	0.00	1.21 (0.26-5.61)		
Cox-Model 2[d]	0.00	1.19 (0.26-5.50)		
Cox-Model 3[e]	0.00	0.90 (0.18-4.40)		
**RAR (Romania) n=30,813**				
Number of revisions	55	173	410	471
Cox-Model 1[c]	**2.06 (1.20–5.53)**	**1.71 (1.27–2.31)**	**1.43 (1.18–1.77)**	**1.41 (1,18–1.69)**
Cox-Model 2[d]	**2.14 (1.24–3.70)**	**1.65 (1.22–2.24)**	**1.32 (1.08–1.61)**	**1.31 (1.09–1.59)**
Cox-Model 3[e]	**2.12 (1.23–3.66)**	**1.70 (1.25–2.31)**	**1.30 (1.07–1.59)**	**1.30 (1.08–1.56)**
**SIRIS (Switzerland) n=93,463**				
Number of revisions	460	1,475	4,537	
Cox-Model 1[c]	1.59 (0.92-2.76)	**2.01 (1.52-2.65)**	**1.71 (1.44-2.02)**	
Cox-Model 2[d]	1.58 (0.91-2.75)	**1.95 (1.48-2.57)**	**1.64 (1.39-1.95)**	
Cox-Model 3[e]	**1.99 (1.10-3.60)**	**2.16 (1.60-2.92)**	**1.84 (1.53-2.20)**	

CI=confidence interval. HR=hazard ratio.

[a] ALCB was the reference group. Bold HR (95% CI) indicates significance with P-value <0.05.
[b] FAR, NAR, PABZ, and SAR were not evaluated in the meta-analysis as there were <100 TKA where plain bone cement was used.

c Cox-model 1 refers to unadjusted model.
d Cox-model 2 refers to model adjusted for age (<55, 55-64, **65-74**, >74), sex (**male**, female), and time period (**2010-2014**, 2015-2020).
e Cox-model 3 refers to fully adjusted model for age (<55, 55-64, **65-74**, >74), sex (**male**, female), time period (**2010-2014**, 2015-2020), ASA (**1**, 2, 3, ≥4), BMI (<18.5, **18.5-24.9**, 25.0-29.9, 30.0-34.9, 35.0-39.9, ≥40), patella resurfacing (**yes**/no), fixation (**fully cemented**, hybrid [cemented tibia], inverse hybrid [cemented femur]), systemic antibiotic prophylactic administered (**yes**, no, unknown), bearing mobility (**fixed**, mobile), and stability (**minimally stabilized**, posterior stabilized). Bold text indicates reference for covariates in the models.
[f] Plain bone cement was used only in 180 TKA and follow-up was less than 5 years.

AOANJRR	= The Australian Orthopaedic Association National Joint Replacement Registry
DKR	= The Danish Knee Arthroplasty Registry
EPRD	= The German Arthroplasty Registry
FAR	= The Finnish Arthroplasty Register
KP	= Kaiser Permanente Total Joint Replacement Registry
LROI	= Dutch Arthroplasty Register
NAR	= The Norwegian Arthroplasty Register
NJR	=The National Joint Registry
NZJR	= The New Zealand Joint Registry
PABZ	= The Bolzano provincial register of knee prostheses- (Autonomous Province of Bolzano-Italy)
PATN	= The Trento provincial register of knee prostheses- (Autonomous Province of Trento-Italy)
RAR	= Romanian Arthroplasty Register
SAR	= The Swedish Arthroplasty Register
SIRIS	= Swiss National Implant Register

eTable 3. Sensitivity analysis of the meta-analyses (Cox-model 2) of revision for PJI and all-causes to determine how sensitive the meta-analyses results are to the results of individual registry contributions.

	**HR (95% CI)**			
	**3 months**	**1 year**	**5 years**	**10 years**
**Registries excluded[a]**	**Revision due to PJI**			
AOANJRR	1.13 (0.74-1.73)	1.13 (0.85-1.51)	1.13 (0.93-1.37)	1.15 (0.95-1.40)
DKR	1.16 (0.79-1.71)	1.16 (0.89-1.51)	1.14 (0.95-1.36)	1.16 (0.97-1.40)
EPRD	1.12 (0.75-1.69)	1.11 (0.84-1.48)	1.09 (0.91-1.32)	1.11 (0.91-1.35)
KP	1.12 (0.72-1.74)	1.13 (0.84-1.51)	1.13 (0.94-1.37)	1.14 (0.93-1.40)
LROI	1.06 (0.73-1.53)	1.13 (0.86-1.48)	1.09 (0.91-1.32)	1.13 (0.93-1.37)
NJR	1.02 (0.68-1.54)	1.03 (0.80-1.32)	1.04 (0.89-1.21)	1.06 (0.90-1.24)
NZJR	1.07 (0.69-1.57)	1.07 (0.80-1.43)	1.11 (0.92-1.35)	1.13 (0.96-1.23)
RAR	0.89 (0.71-1.13)	1.00 (0.81-1.24)	1.04 (0.89-1.21)	1.05 (0.91-1.22)
SIRIS	1.00 (0.68-1.48)	1.09 (0.82-1.44)	1.11 (0.93-1.13)	1.12 (0.94-1.33)
**Registries excluded[a]**	**Revision due to all-causes**			
AOANJRR	0.95 (0.70-1.29)	1.04 (0.86-1.27)	1.02 (0.92-1.14)	1.03 (0.91-1.16)
DKR	0.98 (0.74-1.28)	1.07 (0.90-1.27)	1.05 (0.96-1.13)	1.06 (0.96-1.16)
EPRD	0.93 (0.68-1.26)	1.03 (0.85-1.25)	1.02 (0.92-1.13)	1.03 (0.92-1.16)
KP	0.94 (0.68-1.29)	1.04 (0.86-1.27)	1.02 (0.92-1.14)	1.04 (0.92-1.17)
LROI	0.97 (0.76-1.23)	1.05 (0.88-1.26)	1.02 (0.92-1.14)	1.04 (0.93-1.17)
NJR	0.93 (0.69-1.25)	0.98 (0.82-1.15)	0.99 (0.90-1.08)	1.00 (0.90-1.10)
NZJR	0.90 (0.66-1.23)	1.01 (0.83-1.23)	1.01 (0.90-1.12)	1.01 (0.90-1.14)
PATN	0.92 (0.71-1.19)	1.02 (0.86-1.21)	1.01 (0.92-1.11)	1.02 (0.92-1.14)
RAR	0.83 (0.71-0.98)	0.96 (0.83-1.11)	0.99 (0.91-1.08)	1.00 (0.90-1.10)
SIRIS	0.88 (0.68-1.12)	1.01 (0.85-1.21)	1.02 (0.92-1.12)	1.02 (0.92-1.14)

CI=confidence interval. HR=hazard ratio.

[a] One at a time, one registry was excluded from the meta-analysis to see if it changes in the statistical significance.

AOANJRR = The Australian Orthopaedic Association National Joint Replacement Registry
DKR = The Danish Knee Arthroplasty Registry
EPRD = The German Arthroplasty Registry
FAR = The Finnish Arthroplasty Register
KP = Kaiser Permanente Total Joint Replacement Registry
LROI = Dutch Arthroplasty Register
NAR = The Norwegian Arthroplasty Register
NJR =The National Joint Registry
NZJR = The New Zealand Joint Registry
PABZ = The Bolzano provincial register of knee prostheses- (Autonomous Province of Bolzano-Italy)
PATN = The Trento provincial register of knee prostheses- (Autonomous Province of Trento-Italy)
RAR = Romanian Arthroplasty Register
SAR = The Swedish Arthroplasty Register
SIRIS = Swiss National Implant Register

eTable 4. Sensitivity analysis of meta-analyses (Cox-model 3) of revision for PJI and all-causes to determine how sensitive the meta-analyses results are to the results of individual registry contributions.

	HR (95% CI)			
	3 months	1 year	5 years	10 years
**Registries excluded[a]**	**Revision due to PJI**			
AOANJRR	1.24 (0.77-1.99)	1.19 (0.89-1.60)	1.17 (0.96-1.44)	1.17 (0.95-1.45)
DKR	1.26 (0.79-2.03)	1.22 (0.92-1.56)	1.21 (1.00-1.47)	1.21 (0.99-1.48)
EPRD	1.24 (0.77-1.99)	1.19 (0.88-1.59)	1.17 (0.96-1.44)	1.18 (0.96-1.45)
KP	1.20 (0.71-2.03)	1.19 (0.87-1.62)	1.21 (0.99-1.47)	1.21 (0.99-1.48)
LROI	1.15 (0.74-1.78)	1.19 (0.89-1.58)	1.15 (0.94-1.40)	1.17 (0.95-1.44)
NJR	1.06 (0.66-1.70)	1.07 (0.83-1.37)	1.08 (0.93-1.26)	1.09 (0.92-1.28)
NZJR	1.17 (0.69-1.97)	1.14 (0.83-1.55)	1.19 (0.97-1.46)	1.19 (0.96-1.47)
RAR	0.97 (0.70-1.36)	1.06 (0.83-1.34)	1.11 (0.93-1.32)	1.10 (0.93-1.30)
SIRIS	1.06 (0.67-1.68)	1.14 (0.85-1.52)	1.18 (0.97-1.43)	1.16 (0.97-1.40)
**Registries excluded[a]**	**Revision due to all-causes**			
AOANJRR	1.04 (0.75-1.44)	1.14 (0.88-1.47)	1.11 (0.93-1.33)	1.04 (0.90-1.20)
DKR	1.03 (0.72-1.47)	1.16 (0.91-1.48)	1.14 (0.97-1.34)	1.08 (0.95-1.21)
EPRD	0.99 (0.68-1.44)	1.13 (0.88-1.47)	1.12 (0.95-1.34)	1.06 (0.92-1.21)
KP	0.99 (0.67-1.45)	1.14 (0.87-1.48)	1.12 (0.93-1.34)	1.05 (0.91-1.21)
LROI	1.04 (0.76-1.42)	1.14 (0.89-1.47)	1.11 (0.93-1.33)	1.05 (0.95-1.21)
NJR	0.94 (0.65-1.34)	1.07 (0.83-1.37)	1.07 (0.90-1.27)	0.99 (0.89-1.10)
NZJR	0.85 (0.65-1.40)	1.11 (0.85-1.44)	1.10 (0.92-1.33)	1.03 (0.89-1.19)
PATN	0.97 (0.71-1.34)	1.11 (0.88-1.41)	1.10 (0.93-1.29)	1.04 (0.92-1.17)
RAR	0.89 (0.68-1.18)	1.05 (0.83-1.33)	1.07 (0.90-1.28)	1.01 (0.89-1.14)
SIRIS	0.90 (0.66-1.22)	1.02 (0.84-1.24)	1.03 (0.91-1.15)	1.04 (0.92-1.17)

CI=confidence interval. HR=hazard ratio.

[a] One at a time, one registry was excluded from the meta-analysis to see if it changes in the statistical significance.

AOANJRR = The Australian Orthopaedic Association National Joint Replacement Registry
DKR = The Danish Knee Arthroplasty Registry
EPRD = The German Arthroplasty Registry
FAR = The Finnish Arthroplasty Register
KP = Kaiser Permanente Total Joint Replacement Registry
LROI = Dutch Arthroplasty Register
NAR = The Norwegian Arthroplasty Register
NJR =The National Joint Registry
NZJR = The New Zealand Joint Registry
PABZ = The Bolzano provincial register of knee prostheses- (Autonomous Province of Bolzano-Italy)
PATN = The Trento provincial register of knee prostheses- (Autonomous Province of Trento-Italy)
RAR = Romanian Arthroplasty Register
SAR = The Swedish Arthroplasty Register
SIRIS = Swiss National Implant Register
