# Supplementary material for: Periprosthetic Joint Infection After Total Knee Arthroplasty With or Without Antibiotic Bone Cement
Source: JAMA Netw Open. 2024 May 23;7(5):e2412898. doi: 10.1001/jamanetworkopen.2024.12898 (PMC11117087; doi:10.1001/jamanetworkopen.2024.12898)
Supplement: Supplement 2. — Data Sharing Statement [file jamanetwopen-e2412898-s002.pdf]

Data Sharing Statement

Leta. Periprosthetic Joint Infection After Total Knee Arthroplasty With or Without Antibiotic Bone Cement. *JAMA Netw Open*. Published May 23, 2024.
doi:10.1001/jamanetworkopen.2024.12898

Data
Data available: No

Additional Information
Explanation for why data not available: Due to privacy, security, and data ownership regulation, registries might not share even deidentified patient level data. Thus, accumulative data might be obtained through direct request from each participating registry.
